# Mechanisms of parental distress during and after the first COVID-19 lockdown phase: A two-wave longitudinal study

**DOI:** 10.1371/journal.pone.0253087

**Published:** 2021-06-24

**Authors:** Miriam S. Johnson, Nora Skjerdingstad, Omid V. Ebrahimi, Asle Hoffart, Sverre Urnes Johnson

**Affiliations:** 1 Oslo Metropolitan University, Oslo, Norway; 2 Department of Psychology, University of Oslo, Oslo, Norway; 3 Modum Bad Psychiatric Hospital, Vikersund, Norway; China Agricultural University, CHINA

## Abstract

**Background:**

In these unpredictable times of the global coronavirus disease 2019 (COVID-19) pandemic, parents worldwide are affected by the stress and strain caused by the physical distancing protocols that have been put in place.

**Objective:**

In a two‐wave longitudinal survey, we investigated the levels of parental stress and symptoms of anxiety and depression in a sample of parents at two time points; during the implementation of the strictest physical distancing protocols following the onset of the COVID-19 pandemic (T1, *N* = 2,868) and three months after the discontinuation of the protocols (T2, *N* = 1,489). Further, we investigated the relationships between parental stress and anxiety and depression relative to relationship quality and anger toward their children at the two aforementioned time points, including subgroups based on age, parental role, cultural background, relationship status, education level, number of children, employment status and pre-existing psychiatric diagnosis.

**Methods and findings:**

Parents were asked to fill out a set of validated questionnaires on the two measurement points. Parental stress significantly decreased from T1 to T2, indicating that the cumulative stress that parents experienced during the implementation of the distancing protocols declined when the protocols were phased out. The decrease of perceived parental stress was accompanied by a significant decrease in the symptoms of both depression and anxiety among the participating parents. Symptoms meeting the clinical cut-offs for depression (23.0%) and generalized anxiety disorder (23.3%) were reported among participating parents at T1, compared to 16.8% and 13.8% at T2, respectively. The reduction in depression and anger toward their child(ren) from T1 to T2 was associated with a reduction of parental stress. Relationship quality and anger toward their child(ren) at T1 further predicted a change in the level of parental stress from T1 to T2.

**Conclusions:**

The study underlines the negative psychological impacts of the implementation of the distancing protocols on parents’ health and well-being. Uncovering the nature of how these constructs are associated with parents and families facing a social crisis such as the ongoing pandemic may contribute to the design of relevant interventions to reduce parental distress and strengthen parental coping and resilience.

## Introduction

With the rapid spread of coronavirus disease 2019 (COVID-19), governments worldwide decided to close down schools, kindergartens and workplaces, to slow the spread of the virus, leaving families quarantined in their houses for months. In these unpredictable times of a massive global health crisis, parents and their children are affected by a new range of stressors and strains that challenge their everyday family life and functionality. Although some parents mobilize coping and resilience strategies in the face of the COVID-19 threats [1, 2], some are at risk of experiencing worsened mental health problems or developing new stress-related disorders [3, 4]. The initial evidence obtained indicating how the ongoing pandemic has negatively affected the health and well-being of parents and children [4–10] probably barely scratches the surface of the potential long-term effects of the pandemic on families’ health and well-being. These problems may continue even after the pandemic has abated and the distancing measures have been lifted due to self-sustaining processes.

The unprecedented challenges of experiencing a disruption of one’s daily routines and competing demands on oneself and one’s family, such as working from home while educating and caring for one’s children, unemployment, economic hardship, and worrying about the health of one’s family and relatives, in an environment of physical and social isolation, may exacerbated one’s stress as a parent. Particular concerns have been raised about neglected and vulnerable children, children in at-risk families, and single parents who have been disproportionately affected by the suspension of child-care professionals and support services were suspended during the lockdown [11, 12].

The aforementioned concerns related to the home environment in a social crisis are grounded in the robust literature covering various components of the association between parental stress, mental health problems, and dysfunctional parent–child interactions [13–15]. Parents’ elevated stress levels accompanied by symptoms of anxiety and depression have also been found to negatively affect the parents’ relationships with their children in terms of lower responsiveness to their children’s needs, increased rates of discipline-related confrontation, frequent physical punishment and child abuse and increased child behavioral problems [13, 15–22]. Studies on earlier social crises that examined the levels of distress among quarantined parents reported high levels of parental stress, depression and anxiety [23, 24]. Previous research has also shown that traumatic or life-threatening events, such as natural disasters, may cause the quality of one’s relationships to deteriorate due to stress and conflict [25–27] and increased experiences of domestic violence [28]. On the other hand, various studies have suggested that good relationship quality, may serve as a protective factor that are strongly associated with emotional and psychological well-being and lower perceived stress [25–29].

To date, some cross-sectional studies concerning the different physical distancing measures that have been implemented have provided insight into the impact of the COVID-19 pandemic on the family dynamics and functionality. These studies have investigating how parents’ well-being, as well as mechanisms of parenting, are directly affected by the physical distancing measures related to the pandemic. In a recently published study with a large sample size, the psychological impact of 2-week quarantine on Italian parents was investigated. Clinically alarming levels of distress were reported by 84% of the 1,226 participating parents, and parenting-related exhaustion was reported by 17% [30]. A similar study among 420 American parents conducted 5 weeks after the first quarantined in the United States was advised, reported an average of moderate levels of caregiver burden and scores indicating mild anxiety and below mild depression among the participating parents [31]. A study that examined the impact of the COVID-19 pandemic in relation to parental stress among 183 parents in the western United States reported that parents experienced cumulative stressors due to the COVID-19 lockdown, including general stress and symptoms of depression and anxiety [4]. The results of a study on Iranian parents reported, however, that home quarantine nearly one and a half months after the COVID-19 outbreak did not have a significant effect of parental burnout [32]. Taken together, these results suggest that the lockdown due to the COVID-19 outbreak seems to affect the mechanisms of parental stress and burden of parents differently.

The various findings reported in recent studies may also be reflected by the physical distancing measures implemented, the measurement point during the outbreak, and the representativeness of the samples under study. Cultural and societal differences in terms of divergence in the response to the pandemic may also depend on varying cultural contexts and dimensions of cultural variance, which are important in understanding the divergent responses to the pandemic in general and to the lockdown measures in particular [33, 34].

However, thus far, mainly cross-sectional studies have been carried out to investigate parents’ self-perceived stress levels during the COVID-19 lockdown [4, 35]. Additional longitudinal data, such as those presented in the current study, allow an examination of the changes in parents’ self-perceived stress level and of the predictors of these changes at several time points.

The current study sought to add to the literature on parental stress in a two-wave longitudinal survey. Specifically, the purpose of the present study was to examine the impact of the COVID-19 pandemic on parents’ psychological well-being by investigating the changes in parents’ self-perceived stress level and symptoms of anxiety and depression among parents at two time points: (1) during the implementation of strict government- initiated physical distancing protocols following the onset of the COVID-19 pandemic (T1) and (2) after the discontinuation of the distancing protocols three months later (T2). Furthermore, we examined the relationship between parental stress and symptoms of anxiety and depression, on the one hand, and variables related to family dynamics, such as relationship quality and anger toward their child(ren), on the other hand, at the two aforementioned time points.

First, we hypothesized that there would be a significant decrease in parental stress among parents from T1 to T2. The levels of parent’ self-perceived stress across different demographic subgroups were further investigated.

Second, we hypothesized that higher levels of symptoms of anxiety and depression, lower relationship quality and higher level of anger towards their child(ren), together with less reduction in anxiety and depressive symptoms and less reduction in anger towards child(ren), would be associated with parental stress reduction from T1 to T2, above and beyond the influence of the demographic variables (i.e., parental role, age and the number of children in the household).

## Methods

### Study participants

At T1, the participants included 2,868 parents, 21–83 years of age (*M*_age_ = 39.8; *SD* = 8.0). Most of the participants were female (79.4%; *n* = 2,278) and Norwegian born (91.7%; *n* = 2,631). In terms of family structure, 80.4% (*n* = 2,305) of the parents reported that they were married or cohabiting with a partner and 83.9% of the parents reported living with one child per parent or less in the household. Of the participating parents at T1 (*n* = 2,278 mothers; *n* = 587 fathers), 81.7% of the mothers and 68.5% of the fathers reported having earned a university degree or currently studying. Additionally, 17.2% (*n* = 391) of the mothers and 10.4% (*n* = 61) of the fathers at T1 reported a pre-existing psychiatric diagnosis, reflecting the lower end of the known rate of psychological disorders in Norway’s adult population; 16–25% [36].

At T2, 52% (*n* = 1,489) of the parents who participated at T1 continued to take part in the study. The participants’ ages ranged from 24 to 69 years (*M*_age_ = 40.7; *SD* = 7.3), and most of the participants were female (80.3%; *n* = 1,195) and Norwegian born (92.7%; *n* = 1,381). A high percentage (82.8%; *n* = 1,233) of the parents reported being married or cohabiting with a partner. A high percentage as well (84.5%) reported living with one child per parent or less in the household. Furthermore, of the participants at T2 (*n* = 1,195 mothers, 293 fathers), 85.9% of the mothers and 76.5% of the fathers reported having earned a university degree or currently studying. Moreover, 16.0% (*n* = 191) of the mothers and 9.6% (*n* = 28) of the fathers reported a pre-existing psychiatric diagnosis. Table 1 shows the demographic characteristics of the parental samples at T1 and T2.

**Table 1:** Demographic characteristics of the study sample.

Characteristics	Parents (T1)	Parents (T2)
	(*n* = 2,868)	(*n* = 1,489)
Age	39.8 (*SD* = 8.0)	40.7 (*SD* = 7.3)
Number of child(ren) in household	1.8 (*SD* = 0.8)	1.8 (*SD* = 0.7)
Parental role		
Female	2278 (79.4%)	1195 (80.3%)
Male	587 (20.5%)	293 (19.7%)
Cultural background		
Norwegian	2713 (94.6%)	1410 (94.7%)
1st generation immigrant	127 (4.4%)	66 (4.4%)
2nd generation immigrant	28 (1.0%)	13 (0.9%)
Civil Status		
Single parent	422 (14.7%)	206 (13.8%)
In a relationship	141 (4.9%)	56 (3.8%)
Married/cohabiting	2305 (80.4%)	1227 (82.4%)
Parent work status		
Mother works (yes)	1735 (76.2%)	930 (77.8%)
Father works (yes)	503 (85.7%)	249 (85.0%)

### Recruitment and procedures

The present study has a pre/post- survey design, with the participants asked to fill out a set of validated questionnaires at two measurement points. As further described in the method section, some questionnaires were presented as a whole, whereas other questionnaires consisted of theoretically- driven selections of items from validated questionnaires through a consensus-obtained process involving clinical experts, with the purpose of not overwhelming the participants with a long survey.

The first data collection time point (T1) lasted 7 days, and the data were collected from March 31 to April 7, when psychical distancing protocols were in place in the whole of Norway and had been in place 2 weeks earlier. The physical distancing protocols were identical across all the regions in Norway and included closure of borders, schools/kindergartens/universities, gyms, and all additional businesses involving human contact with increased risk of infection, and cancellation of cultural events. Fourteen-day quarantine was mandatory for individuals who had been in contact with anyone who had been infected, and also for individuals returning to Norway. Isolation was also demanded for anyone with suspected COVID-19 symptoms or who had been confirmed to have the virus. Moreover, individuals were disallowed from meeting in groups of more than five people and had to maintain a distance of at least two metres from others. Traveling to and staying overnight at a leisure property outside the municipality where one resides was also banned. All hospitals and health institutions had to introduce access control and stop regular visitation routines. Health personnel were also disallowed from leaving the country.

No information was provided by the government about possible modifications to any of these protocols during the data collection period, which allowed controlling for expectation effects. All the parents who participated at T1 (*N* = 2,868) were invited to participate in the second wave of data collection (T2), which was conducted from June 22 to July 13. This initiation date for measurement at T2 is exactly two weeks after the government officials announced its upcoming removal of the large number of physical distancing protocols, including closure of schools, kindergartens, and universities, which had been in place since March 12. On June 22, most of the pandemic mitigation protocols that had been in place in Norway were discontinued. From T1 to T2, the corresponding attrition rate for the parent participants was 48.1%. There was no monetary compensation for participating in the study.

To give the parents in Norway equal opportunities to participate in the study, the survey was primarily disseminated through a Facebook for Business algorithm to any adult (i.e., 18 years old or above) residing in Norway. This algorithm disseminates the survey to a random sample of the proportion of the adult population on Facebook (i.e., 85% of the entire adult population of Norway). Seventy percent of all the study participants were recruited through this random selection technique. To reach the residual 15% of adults who were not on Facebook, the survey was systematically disseminated through national, regional, and local platforms (i.e., newspapers, radio stations, and television) across the entire country. Only one of these platforms (i.e., national television) had 1.1 million viewers at the time of broadcast. Thus, with this wide dissemination technique, we estimate having reached the adult population of Norway and having provided all the parents in the country with equal opportunities to participate in the survey. More information regarding the sampling procedure, including sensitivity analysis that was conducted, is provided by Ebrahimi et al. [37].

The study inclusion criteria that were used during the recruitment procedure, were all adults 18 years old or above, currently living in Norway with one or more children under the age of 18 years and thus experiencing identical government-initiated physical distancing protocols, and who had provided digital consent to take part in the study. The exclusion criteria were individuals below 18 and adults not residing in Norway during the measurement period. The stopping rule for data collection was designed to ensure that government-initiated physical distancing protocols were in place held across all the counties of Norway 2 weeks before and in the week of the data collection, and to control for expectation effects by stopping the data collection instantly once information concerning the modification of the government-initiated protocols was obtained. Thus, the stopping rule involved ending the data collection immediately if the government-initiated physical distancing protocols were changed or if new information about forthcoming modifications was given [37]. As the survey was administered online to a random selection of adults, it was not possible for us to stratify beforehand variables such as parental role and the proportions of the participants with a particular marital status and education level. Consequently, we conducted post-stratification.

Data reported in this study is part of a longitudinal research project (the Norwegian COVID-19, Mental Health and Adherence Project), that investigating the psychological impact of the COVID-19 pandemic on the Norwegian population during the period of implementation of the strict government-initiated physical distancing protocols related to the COVID-19 pandemic (T1) and three months after the breakout of the pandemic (T2). The differences between the T1 and T2 measurements is on account of the discontinuation and reduction of the overall number of physical distancing protocols from T1 to T2, with some mitigation protocols being terminated and others being softened. The pre-registered protocol for the present study can be found at Clinicaltrials.gov (Identifier: NCT04442308). Ethical approval of the study was granted by The Regional Committee for Medical and Health Research Ethics (reference number 125510) and The Norwegian Center for Research Data (Ref. No. 802810).

### Measures

#### Demographic background

The parental role, age, cultural background, civil status, education level, and employment status, the number of children in the household, and whether the participants had a pre-existing psychiatric diagnosis were assessed.

#### Self-perceived parental stress

Parent- perceived parental stress was measured using the 18-item Danish Parental Stress Scale (DPSS) [38]. DPSS is divided into two subscales: nine items measuring parental stress (e.g., “I feel overwhelmed by the responsibility of being a parent”) and seven items aimed at measuring the lack of parental satisfaction (e.g., “I enjoy spending time with my children”). Three items from the parental- stress subscale were chosen by a panel of clinical experts to prevent topological overlapping and so as not to overburden the participants. The items that were included were: (1) “I feel overwhelmed by the responsibility of being a parent”; (2) “The major source of stress in my life is my child(ren)”; and (3) “It is difficult to balance different responsibilities because of my child(ren)”. The participating parents reported the extent to which they agreed with the three items in the past month on a 5-point Likert scale ranging from 1 (*“*strongly disagree*”)* to 5 (“strongly agree”). DPSS has shown good convergent and divergent validity in prior samples [38]. In the dataset in this study, the DPSS had good internal reliability, with a 0.81 Cronbach’s alpha at T2 and 0.78 at T1.

#### Depression

The participating parents’ depressive symptoms were assessed with the nine-item Patient Health Questionnaire (PHQ-9) [39]. This measure is routinely used to assess the symptoms of depression in accordance with the diagnostic criteria for major depression disorder, and consists of nine items, each scored on the basis of a 4-point Likert scale ranging from 0 (*“*not at all*”)* to 3 (“almost every day”). The higher scores indicate more symptoms of depression, and 10 is the cut-off point for having symptoms associated with a major depressive disorder of moderate degree. Research supports the validity of the questionnaire for measuring symptoms of depression in the general population [40].

#### Anxiety

Generalized Anxiety Disorder 7 (GAD-7) [41] was used to assess the symptoms of anxiety and worry. It encompasses seven items scored on the basis of a 4-point Likert scale, ranging from 0 (“not at all*”*) to 4 (“almost every day*”*). The scale has been proven to be a valid and reliable measure of anxiety symptoms in the general population [41] and a score 10 was used as the cut-off.

#### Relationship satisfaction

The participating parents’ satisfaction with their relationship was measured by asking respondents whether they “were more content with their relationship since the pandemic outbreak*”* (in Norway: mid-March).

#### Anger toward one’s child(ren)

The participating parents’ anger toward their child(ren) was measured by asking respondents if they during the last two weeks “had been angrier and more frustrated then usual at their child(ren).

### Statistical analysis

Repeated surveys like those in this study typically have many subject dropouts and missing data. As such, the data obtained in this study were analyzed using mixed models with maximum likelihood estimation, which is the state-of-the-art approach for handling missing data [42]. Particularly if there are random missing data, which was likely in the survey in this study, mixed models yield more unbiased results compared to the other analysis methods [43]. Data are said to be missing at random (MAR) when the probability that observed responses are missing depend on the set of observed responses but is unrelated to the specific missing values [42]. This assumption was further strengthened by the result that there were no significant differences between those who responded at T2 and those who only responded at T1 on anxiety (*t* = 0.05, *p* = .99), depression (*t* = -0.08, *p* = .93) and parental stress (*t* = -0.43, *p* = .66) at T1. Even though there could be expected departures from the assumption of MAR, these are rarely serious enough to degrade the performance of maximum likelihood estimation of missing data [42].

Akaike’s information criterion (AIC) was used to compare the fit of one model with that of the others. The models with an AIC reduction greater than 2 were considered better than the other models [44]. In preliminary analysis for parental stress as the dependent variable, a model with a random intercept and a diagonal covariance structure turned out to have the best fit. Inclusion of a random slope did not improve model fit. Assumptions underlying the models: linear relationships, test of homoscedasticity and normal distribution of the residuals were tested.

First, the hypothesis that parental stress would decrease (H1) was tested by using self-perceived parental stress as a dependent variable in a model using time (T1 period = 0; T2 period = 1) as a predictor. Second, demographic variables were added as predictors, but only those demographic variables that were found to be significant were used in the subsequent analyses. Third, the initial (T1) levels of anger toward one’s child(ren), anxiety, and depression were added as predictors. Relationship quality during the whole pandemic period, measured at T2, was considered a constant and was thus included as a predictor. These interactions of the predictor with time represent the tests of H2 regarding the covariates predicting change in parental stress, called Model 1. Finally, the T2 anger toward one’s child(ren), anxiety, and depression as constant covariates were added, together with the interactions of these constant covariates with time. These interactions represent tests of H2 regarding the change in the covariates from T1 to T2, predicting a change in parental stress from T1 to T2, called Model 2.

## Results

The mean level of parental stress among the 2,868 parents who participated in this study was 7.1 (*SD =* 3.2) at (T1) and 6.4 (*SD* = 3.0) at T2 (*n* = 1,489). The demographic information of the participants and the mean level of parental stress in each subgroup at T1 and T2 is shown in Tables 2 and 3, respectively. Paired samples t-test revealed a significant reduction of parental stress from T1 to T2 (*t* = 10.344; *p* < .001). Furthermore, analysis of variance revealed significant differences in the mean level of parental stress between the age groups at T2, and independent t-tests revealed that those being unemployed and those having a pre-existing psychiatric diagnosis had more parental stress at T2.

**Table 2:** Demographic information of the participants at T1 and mean level of parental stress in each subgroup.

Subgroups	*N* (%)	Mean level of parental stress (*SD*)	*p*	*t* or *F*	Cohen’s *d*
All participants		7.1 (3.1)			
Age group, years			< .001	54.05	
18–30	359 (12.5%)	7.3 (3.1)			
31–44	1728 (60.3%)	7.6 (3.2)			
45–64	768 (26.8%)	5.9 (2.8)			
65+	13 (0.45%)	5.6 (2.6)			
Sex			< .001	5.91	0.27
Female	2278 (79.5%)	7.3 (3.2)			
Male	587 (20.5%)	6.4 (2.9)			
Number of children in household			< .001	-4.00	-0.20
< one child per parent	2407 (83.9%)	7.0 (3.1)			
> one child per parent	461 (16.1%)	7.6 (3.2)			
Cultural background			0.65	0.43	
Norwegian	2713	7.1 (3.2)			
First-generation immigrant	127	7.0 (3.0)			
Second-generation immigrant	28	6.6 (2.8)			
Civil Status			0.48	-0.7	-0.03
Single parent	563 (19.6%)	7.0 (3.1)			
Married or cohabiting	2305 (80.4%)	7.1 (3.1)			
Education Level			< .001	-3.47	-0.16
Finished university degree or currently studying	2267 (79.0%)	7.2 (3.2)			
No university degree and not currently studying	601 (21.0%)	6.7 (3.0)			
Currently Employed			0.09	1.70	0.08
Employed	2241 (78.1%)	7.0 (3.1)			
Unemployed	627 (21.9%)	7.3 (3.3)			
Pre-existing psychiatric diagnosis			< .001	-5.41	-0.28
No	2416 (84.2%)	7.0 (3.1)			
Yes	452 (15.8%)	7.8 (3.4)			

**Table 3:** Demographic information of the participants at T2 and mean level of parental stress in each subgroup.

Subgroups	*N* (%)	Mean level of parental stress (*SD*)	*p*	*t* or *F*	Cohen’s *d*
All participants	1489	6.4 (3.0)			
Age group, years			< .001	18.33	
18–30	120 (8.1%)	6.5 (3.0)			
31–44	917 (61.6%)	6.8 (3.1)			
45–64	449 (30.2%)	5.5 (2.7)			
65+	3 (0.2%)	8.3 (4.7)			
Parental role			0.01	2.47	0.16
Female	1195 (80.3%)	6.5 (3.0)			
Male	293 (19.7%)	6.0 (3.0)			
Number of children in household			0.05	-1.97	-0.14
< one child per parent	1258 (84.5%)	6.3 (3.0)			
> one child per parent or disabled child	231 (15.5%)	6.7 (3.2)			
Cultural background			0.20	1.41	
Norwegian	1419	6.4 (3.0)			
First-generation immigrant	60	6.8 (3.4)			
Second-generation immigrant	10	5.6 (2.7)			
Civil Status			0.12	1.56	0.12
Single parent	200 (13.4%)	6.7 (3.1)			
Married or cohabiting	1289 (86.6%)	6.3 (3.0)			
Education Level			0.08	-1.74	-0.12
Finished university degree or currently studying	1252 (84.1%)	6.4 (3.0)			
No university degree and not currently studying	237 (15.9%)	6.1 (3.0)			
Currently Employed			< .001	3.62	0.23
Employed	1180 (79.2%)	6.2 (2.9)			
Unemployed	309 (20.8%)	6.9 (3.3)			
Pre-existing psychiatric diagnosis			< .001	-3.80	-0.28
No	1270 (85.3%)	6.3 (2.9)			
Yes	219 (14.7%)	7.1 (3.3)			

*Note*: The age group above 65 + did only include 3 persons and was not included in the analyses.

Table 4 provides information about the number of parents who met the clinical cut-off for depression and anxiety at T1 and T2. With regard to the reported symptoms of depression (PHQ-9) among the participating parents, 23.0% had symptoms meeting the clinical cut-off for depression at T1, compared to 16.8% at T2. Also, 23.3% had symptoms meeting the clinical cut-off for generalized anxiety disorder (GAD-7), at T1, compared to 13.8% at T2. These findings suggest a significant decline in both the depression symptoms (*t* = 5.72, *p* < .001) and anxiety symptoms (*t* = 8.93; *p <* .001) across the two time points.

**Table 4:** Participants in different subgroups meeting the diagnostic cut-off score for depression and anxiety across time points.

Subgroups	No (%) of participants meeting the diagnostic cut-off at T1 and T2	
**Symptoms of depression (PHQ-9)**	**T1**	**T2**
All participants	659 (23.0)	250 (16.8)
Sex		
Female	577 (25.3)	213 (17.8)
Male	81 (13.8)	37 (12.6)
Age group, years		
18–30	123 (34.3)	33 (27.5)
31–44	420 (24.3)	168 (18.3)
45–64	115 (15.0)	49 (10.9)
65+	1 (7.7)	0
Civil status		
Single parent	150 (35.5)	53 (25.7)
Married or cohabiting	509 (20.8)	197 (15.4)
Number of children in household		
< one child per parent	566 (23.5)	212 (16.8)
> one child per parent or disabled child	93 (20.2)	38 (16.5)
**Symptoms of anxiety (GAD-7)**		
All participants	667 (23.3)	205 (13.8)
Sex		
Female	585 (25.7)	171 (14.3)
Male	82 (14.0)	34 (11.6)
Age group, years		
18–30	122 (34.0)	22 (18.3)
31–44	434 (25.1)	146 (15.9)
45–64	111 (14.4)	37 (8.2)
65+	0 (0.0)	0 (0.0)
Civil status		
Single parent	135 (32.0)	42 (20.4)
Married or cohabiting	532 (21.8)	163 (12.7)
Number of children in household		
< one child per parent	571 (23.7)	171 (13.6)
> one child per parent or disabled child	96 (20.8)	34 (14.8)

### Multilevel models

As hypothesized, there was a significant decrease in the self-perceived parental stress among the participating parents from T1, when the government-initiated physical distancing protocols were in place in all the counties in Norway, to T2, when such physical distancing protocols were discontinued, *B* = -0.72, *SE* = 0.07, *t*(1,707) = -10.87, *p* < .001). The demographic variables age, parental role, and the number of children were entered as predictors of parental stress. The interaction of number of children with time was not related to parental perceived stress; that is, the number of children was not significantly related to the change in the level of parental stress, *B* = -0.10, *SE* = 0.08, *t*(1,691) = -1.28, *p* = .200.

However, the interaction of age with time indicated that a higher age led to less decrease in parental stress, *B* = 0.03, *SE* = 0.01, *t*(1,693) = 2.61, *p* < .01. The interaction of parental role with time was also observed to have a trend, indicating less decrease for the fathers, *B* = 0.36, *SE* = 0.17, *t*(1,693) = 2.13, *p* = 0.034. As only age had a significant interaction with time at our pre-defined significance level (*p <* .01), only age was used as a control variable in the subsequent analyses.

Table 5 presents the results of the T1 predictors. As shown in Model 1 and as hypothesized, the interaction of relationship quality and anger toward one’s child(ren) at T1 with time predicted the level of parental stress. However, the level of anxiety and depression at T1 was not related to a change in the level of parental stress. As shown in Model 2 and as hypothesized, depression and anger toward one’s child(ren) at T2 predicted less reduction of parental stress. Thus, the reduction of these variables from T1 to T2 is associated with a reduction in perceived parental stress. This pattern, however, was not found for anxiety.

**Table 5:** Predictors of parental stress.

Predictor	Estimate	SE	t	Estimate	SE	t
	Model 1			Model 2		
Intercept	4.45	0.87	5.12*	5.57	0.91	6.14*
	[2.74, 6.15]			[3.79, 7.34]		
Time	1.03	0.54	1.92*	-0.77	0.53	-1.47
	[-0.02, 2.08]			[-1.80, 0.25]		
Age	-0.06	0.02	-3.69*	-0.07	0.02	-3.94*
				[-0.09, -0.03]		
	[-0.09, -0.03]					
Relationship	0.13	0.11	1.12	0.01	0.12	0.07
	[-0.10, 0.36]			[-0.22, 0.24]		
Depression^1^	0.15	0.04	3.96*	0.21	0.04	4.99*
				[0.13, 0.30]		
	[0.08, 0.22]					
Anxiety^1^	-0.06	004	-1.44	-0.04	0.05	-0.87
				[-0.13, 0.05]		
	[-0.15, 0.02]					
Angry-child^1^	1.89	0.10	19.55*	2.05	0.11	19.28*
				[1.84, 2.25]		
	[1.70, 2.08]					
Time X age	0.01	0.01	1.04	0.02	0.01	2.02
				[0.01, 0.04]		
	[-0.01, 0.03]					
Time X Relationship	-0.24	0.07	-3.37*	-0.06	0.07	-0.93
				[-0.19, 0.07]		
	[-0.38, -0.10]					
Time X Depression^1^	-0.06	0.02	-2.39	-0.12	0.03	-4.59*
				[-0.16, -0.07]		
	[-0.10, -0.01]					
Time X Anxiety^1^	0.07	0.03	2.41	0.03	0.03	1.20
				[-0.02, 0.09]		
	[0.01, 0.12]					
Time X Angry-child^1^	-0.60	0.06	-10.10*	-0.87	0.06	-14.29*
				[-0.99, -0.75]		
	[-0.72, -0.48]					
Depression^2^				-0.10	0.05	-2.15
				[-0.2, -0.01]		
Anxiety^2^				-0.01	0.06	-0.07
				[-0.1, 0.1]		
Angry-child^2^				-0.43	0.13	-3.38*
				[-0.69, -0.18]		
Time X Depression^2^				0.08	0.03	2.75*
				[0.02, 0.13]		
Time X Anxiety^2^				0.02	0.03	0.59
				[-0.04, 0.09]		
Time X Angry-child^2^				0.81	+.08	10.76*
				[0.66, 0.96]		

*p = <0.01

^1^ = T1

^2^ = T2, 95% Confidence Interval in brackets.

## Discussion

The present study investigated changes in the levels of parents’ self-perceived stress and symptoms of depression and anxiety during the implementation of the strictest physical distancing protocols (T1), compared to after such protocols were discontinued (T2). As expected, the level of parental stress significantly decreased from T1 to T2, indicating that the parents’ overall stress declined when the physical distancing protocols were phased out. The decrease of parental stress at the two time points was accompanied by a significant decrease in the symptoms of both depression and anxiety.

The decrease in self-perceived parental distress from T1 to T2 may appear as a function of several factors associated with the repeal of the stringent lockdown restrictions. At T2, normal everyday life had started to return. Various significant resources for families re-opened after the lockdown period, including support services, community-level resources, and day care centers and schools, which are also significant recovery environments for children [45]. As the lockdown restrictions were beginning to ease, businesses started to re-open, and people started returning to work. Various studies have found a significant protective effect of employment on mental health in general and on depression and psychological distress in particular [46]. Employment has been found to be associated with improved self-esteem, greater well-being, increased social contact, and independence, all of which contribute to good mental health [46]. Work is also an important social arena and vehicle for social interaction and support, playing a beneficial role in the maintenance of psychological well-being [47]. After months with minimal human interaction, a more balanced life, including meeting family and friends and engaging in social and recreational activities, may have contributed to the decrease in self-perceived parental distress among the parents who participated in this study. This finding is in line with those of earlier studies suggesting that perceived control and balance amidst stressful life events are associated with psychological well-being [23, 48] and decrease in overall stress, anxiety, and depression [49, 50]. Moreover, coping with stress may be easier when the quarantine duration is no longer uncertain and when the perceived controllability related to the quarantine is restored despite the fact that other circumstances regarding the pandemic are not yet illuminated.

It is important to note that not all the parents who participated in this study reported experiencing high levels of parental stress during the implementation of the strictest physical distancing protocols, although the protocols were collectively experienced by the parents. This finding is in line with the existing evidence suggesting that not everyone is at risk of developing higher stress even though everyone is exposed to the same stressful life events [51]. Individuals appraise events differently, and individual responses to such events vary according to individuals’ subjective perceptions of the stressors and different coping strategies [51, 52]. According to Lazarus and Folkman [51], this appraisal involves estimating the resources available and the most effective strategies for dealing with the situation, and a key element of such appraisal is the extent to which the individual can maintain control over the outcome of the situation. Additionally, a range of protective factors may serve as a buffer for families experiencing stressful events [53].

Indeed, the results of this study support the aforementioned notions because the participating parents’ relationship satisfaction was found to predict a change in the level of parental stress. This finding is in accordance with the vast literature that highlights relationship quality and satisfaction as significant protective factors. Several studies have indicated that relationship satisfaction and a supportive family environment are strongly associated with individual psychological well-being and lower levels of stress (see [29] for a meta-analysis on this topic). Relationship satisfaction is also considered a vital source of emotional and psychological well-being when families experience a social crisis, such as the ongoing pandemic [27]. Some studies have also indicated that highly stressful life events such as natural disasters may increase relationship quality [25, 26].

Interestingly, the participating parents’ anger toward their child(ren) at T1 was found to predict a change in the level of self-perceived parental stress. This finding is in line with those of previous studies where stress stemming from parent-child interaction was examined, specifically in relation to anger expression. Increased parental stress has repeatedly been identified as a risk factor for maladaptive parenting practices (see [54] for at review). A predominant finding in several studies is that parental-stress level is associated with abusive parenting [55] and anger expression toward one’s children [56, 57]. For example, Rodriguez and Green [55] found that parental stress and anger expression were correlated with child abuse potential. Interestingly, the two factors combined further predicted the child abuse scale scores, indicating that both stress and anger expression toward one’s children was found to significantly contribute to the level of parental stress [55]. Another study reported that anger expression and parental stress were found to be predictive of child abuse potential and physical aggression toward children [57].

However, studies that indicated a robust link between the levels of parental stress and anger expression are accompanied by studies that showed that child anger proneness and child emotion dysregulation predict parental stress [58]. These findings are particularly interesting when seen in the light of stressful life events such as the ongoing pandemic, where child anger proneness and child disruptive- behavior problems may be children’s reactions to the physical distancing protocols in place [59], which in turn may contribute to the patterns seen in recent and in the present study. The association between self-perceived stress and anger need not be considered unidirectional, as expressions of anger may create stressful situations and vice versa. This mechanism may also be present in the finding, which indicates that the reduction in both anger towards one’s child(ren) and depression from T1 to T2 were associated with a reduction of the self-perceived parental stress. This finding underline the fact that parental stress may be driven by several possible mechanisms, including parental depression [22].

### Strengths and limitations

A major strength of this study is that it captured the detrimental effects of the government-initiated physical distancing protocols applied globally, making the study’s findings generalizable across similar cultures employing similar physical distancing protocols. Another strength of the present study is the large sample of parents experiencing identical interventions across the two measurement periods. Although the sample consisted of both men and woman, there were more (and more well-educated) female participants than male participants, which may introduce a bias in the sample although the sensitivity analysis showed robust results [37]. A limitation of this study is that it is based on self-reported measures. Further, some measures (i.e., relationship satisfaction and anger towards one’s children) are based on unvalidated single items. Although more robust full-scale measures would capture these variables more sufficiently, single items are useful for capturing specific constructs without compromising practical constraints, including survey length and respondent burden, which again may compromise the sample size.

## Conclusions

Overall, the findings of this study underline some of the negative psychological impacts of the physical distancing protocols on parents’ health and well-being. As expected, the levels of parental stress and symptoms of depression and anxiety symptoms in the parental sample significantly decreased when the physical distancing protocols were phased out. These results support the earlier findings that quarantine increases susceptibility to stress and may have harmful effects on mental health, as seen in the general population [23, 60–62] and in particularly in parents [4, 24, 35]. Our findings are also consistent with those of recently published studies, that the parents experienced cumulative stressors due to COVID-19 and that a majority of the parents reported experiencing symptoms of anxiety and depression [4]. Parents who are faced with the competing demands of limiting their social interaction and remaining at home with their children may be particularly vulnerable to psychological distress as an important side effect of mass quarantine. With regard to the future research, multiple time points are needed to unlock the patterns between parental- stress level and factors such as relationship quality and anger expression. Uncovering the nature of how these constructs are associated with a social crisis in particular, can contribute to the design of relevant interventions to reduce parental stress.

## Supporting information

S1 Appendix (PDF)
