# Supplementary material for: Mechanisms of parental distress during and after the first COVID-19 lockdown phase: A two-wave longitudinal study
Source: PLoS One. 2021 Jun 24;16(6):e0253087. doi: 10.1371/journal.pone.0253087 (PMC8224894; doi:10.1371/journal.pone.0253087)
Supplement: S1 Appendix — (PDF) [file pone.0253087.s001.pdf]

Appendix X

Demographic information

How old are you? (in numbers) _____

What is your biological sex?

- o Female
- o Male
- o Transgender
- o Intersex

Is your self-identified gender the same as your biological sex?

- o Yes
- o No

What is your civil status?

- o Single
- o Married or cohabitant
- o In a relationship

What is your current level of education?

- o Completed primary school
- o Completed high school
- o Currently a student at a university/college
- o Completed college/university degree
- o Not completed primary school

Please enter your cultural background

- o Born in Norway
- o European background
- o Middle East or North Africa
- o Mid-Asian
- o East-Asian and South-East-Asian
- o African background except North Africa
- o Oceanian background
- o North American background
- o Latin-American and Caribbean background

Are you a refugee or immigrant?

- o Yes
- o No

What is your current work situation?

- o Full-time job
- o Sick leave
- o AAP (Work clearance allowance)
- o Disability benefit
- o Retired
- o Student

- Stay-at-home
- Part time job
- Parental leave
- Other

Do you have a current psychological disorder?
- Yes
- No

Do you have children?
- Yes
- No

If yes How many children do you have?
- 1
- 2
- 3
- 4
- 5 or more

How many children under 18 years do you live with?
- No one
- 1
- 2
- 3
- 4
- 5 or more
- I am (responsible) for a disabled child

Parental Stress/anger at child

How much have the following statements applied to you during the last two weeks?

"I feel overwhelmed by the responsibility of being a parent,"

"The major source of stress in my life is my children."

"It is difficult to balance different responsibilities because of my children."

"I have been angrier and more frustrated then usual at my children".

The item was scored on a five-point Likert-Scale ranging from Strongly disagree (1) to Strongly agree (5).

Depression – PHQ-9

How often have you been bothered by the following over the past two weeks?

"Little interest or pleasure in doing things?"

"Feeling down, depressed, or hopeless?"

"Trouble falling or staying asleep, or sleeping too much?"

"Feeling tired or having little energy?"

"Poor appetite or overeating?"

"Feeling bad about yourself — or that you are a failure or have let yourself or your family down?"

"Trouble concentrating on things, such as reading the newspaper or watching television?"

"Moving or speaking so slowly that other people could have noticed? Or so fidgety or restless that you have been moving a lot more than usual?"

"Thoughts that you would be better off dead, or thoughts of hurting yourself in some way?"

The items were scored on a four-point Likert-Scale ranging from Not at all (0) to almost every day (3).

Anxiety – GAD-7

Over the last 2 weeks, how often have you been bothered by any of the following problems?

"Feeling nervous, anxious or on edge?"

"Not being able to stop or control worrying?"

"Worrying too much about different things?"

"Trouble relaxing?"

"Being so restless that it is hard to sit still?"

"Becoming easily annoyed or irritable?"

"Feeling afraid as if something awful might happen?"

The items were scored on a four-point Likert-Scale ranging from Not at all (0) to almost every day (3).

Relationship satisfaction

"Since the pandemic outbreak (Middle of March) Have I been more satisfied with my relationship"

The item was scored on a five-point Likert-Scale ranging from Not more than usual (1) to much more than usual (5).
